# Supplementary material for: Deep and Frequent Phenotyping study protocol: an observational study in prodromal Alzheimer’s disease
Source: BMJ Open. 2019 Mar 23;9(3):e024498. doi: 10.1136/bmjopen-2018-024498 (PMC6475176; doi:10.1136/bmjopen-2018-024498)
Supplement: Supplementary file 1 [file bmjopen-2018-024498supp001.pdf]

The authors of the Deep and Frequent Phenotyping protocol would like to express their gratitude to the following individuals who contributed to the study's preparatory work:

Sites

Oxford

Jasmine Blane (Research Assistant, Department of Psychiatry, University of Oxford)

Amy Chinner (Research Assistant, Department of Psychiatry, University of Oxford)

Sophie Walker (Research Assistant, Department of Psychiatry, University of Oxford)

Leona Wolters (Research Assistant, Department of Psychiatry, University of Oxford)

Manchester

Angela Parker (Research Delivery Manager NIHR CRN: Greater Manchester/University of Manchester)

Gemma Stringer (Research Assistant, University of Manchester)

Tobias Langheinrich (Principal Investigator, University of Manchester)

Newcastle

Katherine Walker (Research Nurse, Newcastle University)

Ginette Cass (Medical Registrar, Newcastle University)

King's College London

Carol Bannister (Sub-Investigator, King's College London)

Exeter

Anna Steele (Senior Research Nurse, NIHR Exeter Clinical Research Facility)

Robert Wells (Clinical Research facilitator, NIHR Exeter Clinical Research Facility)

Kim Gooding (Research Fellow, University of Exeter Medical School)

Edinburgh

Sarah Gregory (Study Coordinator, Centre for Dementia Prevention, University of Edinburgh)

Laura Doull (Clinical Project Manager, Centre for Dementia Prevention, University of Edinburgh)

Fiona Gunn (Study Coordinator, Centre for Dementia Prevention, University of Edinburgh)

West London NHS Trust / Imperial College London

Genevieve Morrison (Research Delivery Service Manager, West London NHS Trust)

Modalities

Cognition

Francesca Cormack (Researcher, Cambridge Cognition, Cambridge)

Charlotte Housden (Researcher, Cambridge Cognition, Cambridge)

Jennifer Barnett (Researcher, Cambridge Cognition, Cambridge)

Elizabeth Baker (Researcher, Cambridge Cognition, Cambridge)

PET and MRI Imaging

Azadeh Firouzian (Post-doctoral Researcher, Invicro, London)

Johannes Klein (Consultant Neurologist, PET lead Oxford)

Nicola Filippini (MRI Manager, OHBA, Oxford)

William Hallet (Head of Imaging Physics, Invicro, London)

Laura Parkes (Senior Lecturer, University of Manchester)

Julian Matthews (Senior Lecturer, University of Manchester)

Neurophysiology

Rik Henson (Deputy Director, MRC Cognition and Brain Sciences Unit, University of Cambridge)

Laura Hughes (Post-doctoral researcher, MRC Cognition and Brain Sciences Unit, University of Cambridge)

Alicia Wilcox (Doctoral researcher, MRC Cognition and Brain Sciences Unit, University of Cambridge)

Rebecca Beresford (Doctoral researcher, MRC Cognition and Brain Sciences Unit, University of Cambridge)

Molecular biomarkers

Henrik Zetterberg (Professor of Neurochemistry, Sahlgrenska Academy, University of Gothenburg, Sweden)

Ophthalmological assessment

Tunde Peto (Professor in clinical Ophthalmology, Ophthalmology image analysis lead, Queen's University Belfast)

Lajos Csincsik (Doctoral student; Ophthalmology modality manager, Queen's University Belfast)

Alyson Muldrew (Research Fellow, Imaging analyst, Queen's University Belfast)

Barbara Hamill (Ophthalmic Science Practitioner, Queen's University Belfast)

Peter Issa (Professor in Ophthalmology, Ophthalmology lead Oxford)

Alexina Fantato (Clinical Research Nurse Manager, Oxford Eye Hospital)

Humma Shahid (Consultant Ophthalmic Surgeon, Ophthalmic site lead, Addenbrooke's Hospital Cambridge)

Keith Martin (Professor of Ophthalmology, Addenbrooke's Hospital Cambridge)

Liz Orlowski (Clinical Trial Coordinator, Addenbrooke's Hospital Cambridge)

Poonam Shah (Research Optometrist, Addenbrooke's Hospital Cambridge)

Kim Baxter (Ophthalmic Photography Team Leader, Addenbrooke's Hospital Cambridge)

Haralabos Eleftheriadis (Consultant Ophthalmic Surgeon Ophthalmology site lead, King's College Hospital London)

Noimot Timson (Senior Research Coordinator, King's College Hospital London)

Richard Leung (Ophthalmic Science Practitioner, King's College Hospital London)

Chandra Devkota (Ophthalmic Science Practitioner, King's College Hospital London)

Stefanos Efraimidis (Medical Retina Specialist, King's College Hospital London)

Christina Granavou-Xirou (Research Fellow, King's College Hospital London)

Yvonne D'sousa (Consultant Ophthalmologist, Ophthalmology lead, Manchester Royal Eye Hospital)

Jane Gray (Head of Diagnostics Ophthalmic Angiography & Imaging Services, Manchester Royal Eye Hospital)

Stuart Hesketh (Ophthalmic Science Practitioner, Manchester Royal Eye Hospital)

Mark Jotham (Ophthalmic Science Practitioner, Manchester Royal Eye Hospital)

Luke Carine (Ophthalmic Science Practitioner, Manchester Royal Eye Hospital)

William Innes (Consultant Ophthalmologist, Ophthalmology site lead, Newcastle Eye Center)

Karl Southerton (Ophthalmic Science Practitioner, Newcastle Eye Center)

Richard Bell (Ophthalmic Science Practitioner, Newcastle Eye Center)

Tom MACGILLIVRAY (Research Fellow, Image Analysis Core Laboratory Manager, Ophthalmology lead, The University of Edinburgh)

Kirstie HETHERINGTON (Photographer, The University of Edinburgh)

Angela Shore (Professor of Cardiovascular Sciences and Scientific Director of the NIHR Exeter Clinical Research Facility, Ophthalmology site lead, University of Exeter Medical School)

Wearable and digital devices

Silvia Del-Din (Post-doctoral research, University of Newcastle)

Chris Hinds (Mezurio app lead investigator, Big Data Institute, Oxford)

Claire Lancaster (Post-doctoral researcher, Big Data Institute, Oxford)

Niki Trigoni (Lead investigator indoor localisation system; Professor of Computing Science, Department of Computer Science, University of Oxford)

Stefano Rosa (Doctoral student, Department of Computer Science, University of Oxford)

Cecilia Mascolo (Lead investigator outdoor navigation monitoring app; Professor of Mobile Systems in the Department of Computer Science and Technology, University of Cambridge)

Dionysis Manousakas (Doctoral student, Department of Computer Science and Technology, University of Cambridge)

Sandra Servia (Post-doctoral researcher, Department of Computer Science and Technology, University of Cambridge)

John Ainsworth (Professor of Health Informatics, The University of Manchester)

Mathew Machin (mHealth Applications Manager, The University of Manchester)
