# Supplementary material for: Deep and Frequent Phenotyping study protocol: an observational study in prodromal Alzheimer’s disease
Source: BMJ Open. 2019 Mar 23;9(3):e024498. doi: 10.1136/bmjopen-2018-024498 (PMC6475176; doi:10.1136/bmjopen-2018-024498)
Supplement: Supplementary file 2 [file bmjopen-2018-024498supp002.pdf]

## Inclusion Criteria

1. Men and women aged 60 years and above, at baseline on Day 1.
2. Family history of dementia in first degree relatives, to be reviewed by the study investigator.
3. Participants must understand the nature of the study and must provide signed and dated written informed consent prior to initiation of any study-related procedures. Participants who are deemed incapable of providing informed consent may not be enrolled.
4. Cerebral amyloid status as determined by PET or CSF Aß, in accordance with study recruitment requirements (4:1 ratio of amyloid positive to negative in final study population).
5. Rosen Modified Hachinski Ischaemic score of ≤ 4.
6. The participant should be mentally and physically able to understand and participate in all scheduled assessments and to complete all required tests and procedures, as judged by the Investigator, including PET imaging, lumbar punctures and computerised assessments.
7. Able to read and write and with minimum 7 years of formal education (full time school college, university, but not including vocational training schemes and manual apprenticeships).
8. The participant must be able to lie comfortably on their back for up to 90 minutes at a time.
9. The participant must be able to walk independently for at least 10 metres, without a walking aid.

## Exclusion Criteria

1. Significant neurological disease affecting the CNS, other than prodromal AD, that may affect cognition or ability to complete the study, including, but not limited to: other dementias, serious infection of the brain, epilepsy or Parkinson's disease.
2. CDR ≥ 1
3. Any clinically important abnormality as determined by the Investigator at screening or between screening and Day 1, in medical history, physical examination, neurological examination (including significant focal findings), vital signs or clinical laboratory test results that could be detrimental to the participant or could compromise the study.
4. Presence of any significant psychiatric disorder, according to the criteria of the Diagnostic and Statistical Manual of Mental Disorders, 5$^{th}$ Edition – Text Revision (DSM-V), or symptom, if, in the judgement of the Investigator, the psychiatric disorder or symptom is likely to confound interpretation of cognitive assessment or affect the participant's ability to complete the study. Participants with mild affective disorder may be included if, in the judgment of the investigator, the disorder is not sufficient to explain the cognitive deficits.
5. Medications with the potential to significantly affect cognition, including but not limited to: anxiolytics, antidepressants, antipsychotics and sedative-hypnotics, are not permitted unless maintained on a stable dose regimen for at least 30 days prior to Day 1.

6. Impairment of vision or hearing that would make cognitive testing, MEG or EEG testing difficult, as judged by the Investigator. Neither hearing aids nor standard glasses can be worn in the MEG system. Hearing frequencies at certain frequencies may be tested using a brief auditory threshold paradigm. Each MEG centre will inform the Investigator of the range of optical prescriptions that can be satisfactorily corrected using MEG-compatible glasses.
7. History of multiple episodes of minor head trauma or any head trauma resulting in protracted loss of consciousness, or serious infectious disease affecting the brain, within five years of screening or between screening and Day 1.
8. History of seizure, except febrile seizures or single provoked seizure.
9. A clinically significant illness (including chronic, persistent or acute infection), medical or surgical procedure, or trauma within 30 days prior to screening or between screening and Day 1.
10. Current serious or unstable clinically important illness, including hepatic, renal, gastroenterologic, respiration, cardiovascular, endocrinologic, immunologic, haematologic or other major disease, that in the judgment of the Investigator, is likely to deteriorate or affect the participant's safety or ability to complete the study.
11. Known or suspected systemic infection e.g. human immunodeficiency virus (HIV), hepatitis B virus (HBV) or hepatitis C virus (HCV).
12. Poor venous access or other contraindications that would make blood sampling difficult.
13. Any spinal malformations, other clinical findings (e.g. papilloedema seen with ophthalmoscopy, coagulation defect, symptoms of increased intracranial pressure) or other aspects (e.g. tattoos) that may complicate or contraindicate lumbar puncture, as judged by the Investigator.
14. Patients with claustrophobia or any other condition that would make the participant unable to undergo an MR or PET scan.
15. A contraindication for an MRI, including but not limited to: MR-incompatible pacemakers, pregnancy, aneurysm clip, implanted neural stimulator, implanted cardiac pacemaker or auto-defibrillator, cochlear implant, ocular foreign body, recent carotid stent, CSF shunt, other implanted medical device (e.g. Swan Ganz catheter, insulin pump), as assessed by a standard pre-MRI questionnaire.
16. Participation in a research study or other radiation exposure, which in conjunction with this study, would result in additional ionising radiation exposure exceeding 10mSv within a 12 month period.
17. Participation in a Clinical Trial of an Investigational Product (CTIMP) in the last 30 days (continued participation in the parent cohort is expected). Participation in a non-CTIMP is not an exclusion criterion, unless in the opinion of the Investigator it would compromise study procedures.
18. Metallic implants in the body with a likelihood of producing excessive artefacts in the MEG scanner. This may include metallic implants which would not exclude a participant from a MRI scan e.g. plates or screws in bone. Whether the metal implant will cause a problem depends largely on where it is in the body (e.g. hip and knee replacements are generally acceptable, but shoulder or cranial implants are not). The Investigator will be advised by experienced researchers at each MEG centre. If it is unclear whether the participant will be

suitable, then they may be invited to the MEG centre for an artefact test prior to participation.

19. Inability to sit at a retinal camera.
20. A diagnosis of iris clip lens.
21. Untreated angle closure glaucoma.

    Any other reason deemed likely to significantly affect compliance with
